# Resveratrol Inhibits Propagation of* Chlamydia trachomatis* in McCoy Cells

**DOI:** 10.1155/2017/4064071

**Published:** 2017-11-29

**Authors:** Ivan M. Petyaev, Nayilia A. Zigangirova, Elena Y. Morgunova, Nigel H. Kyle, Elena D. Fedina, Yuriy K. Bashmakov

**Affiliations:** ^1^Lycotec Ltd., Granta Park, Cambridge CB21 6GP, UK; ^2^Department of Medical Microbiology, Gamaleya Federal Research Center of Epidemiology and Microbiology, Ministry of Health, 18 Gamaleya Str., Moscow 123098, Russia

## Abstract

Resveratrol (RESV), an antifungal compound from grapes and other plants, has a distinct ability to inhibit the* Chlamydia (C.) trachomatis *developmental cycle in McCoy cells, a classic cell line used for chlamydial research. Inoculation of* C. trachomatis *with increasing amounts of RESV (from 12.5 to 100 *μ*M) gave a dose-dependent reduction in the number of infected McCoy cells visualized by using monoclonal antibodies against chlamydial lipopolysaccharide. A similar trend has been observed with immunoassay for major outer membrane protein (MOMP). Furthermore, there was a step-wise reduction in the number of* C. trachomatis *infective progenies caused by the increasing concentrations of RESV. The ability of RESV to arrest* C. trachomatis *growth in McCoy cells was confirmed by a nucleic acid amplification protocol which revealed dose-dependent changes in mRNAs for different genes of chlamydial developmental cycle (*euo*,* incA*, and* omcB*). Although the precise nature of the antichlamydial activity of RESV is yet to be determined and evaluated in future studies, the observed effect of RESV on* C. trachomatis *infection was not related to its potential effect on attachment/entry of the pathogen into eukaryotic cells or RESV toxicity to McCoy cells. Similar inhibitory effect was shown for* C. pneumoniae *and* C. muridarum*.

## 1. Introduction

Resveratrol (3,4′,5-trihydroxystilbene, С_14_Н_12_О_3_, RESV) belongs to a group of polyphenolic substances named stilbenes. Its fat-soluble* cis*- and* trans *isoforms bound to a glucose molecule are present in some plants (grapes, peanuts, berries, etc.) and red wine [1]. Steadily growing interest in the potential health benefits of resveratrol began to surface about 20 years ago when reduction in the risk of cardiovascular disease was linked to moderate red wine consumption [2]. Interest in the potential health benefits of RESV was also fueled by reports relating to the anti-aging properties of the compound when RESV-mediated extension of lifespan in yeast,* C. elegans *and* Drosophila, *was reported [3]. To date, RESV is acknowledged to have significant antioxidant activity and anticarcinogenic as well as cardio- and neuroprotective actions [4]. Reports on the antidiabetic activity of RSV and other plant-derived polyphenols have also emerged recently [5, 6]. The remarkable diversity of biological effects of RESV seen in mammals might be explained by multiple targets mediating its action in intermediate metabolism. Among these are molecules and pathways involved in aging and longevity, mTOR/S6K, sirtuins, AMPK, and perhaps several others [7]. Therefore, the RESV-driven health effects seen in higher organisms are likely to be secondary to the antiaging effect of RESV reported in metazoans. However, it is important to acknowledge that the beneficial effects of RESV reported in eukaryotes have little or no relevance to plant physiology. RESV is a phytoalexin, a normal constituent of the plant cell produced by plants in response to fungal/bacterial insult, stress, or elicitor treatment [7]. Therefore, antifungal and antibacterial properties seem to constitute an original biological function of RESV whose significance for medical practice has not yet been fully explored. Such an assumption is well supported by recent studies reporting the inhibitory effect of RESV on different types of viral infections in eukaryotes [8, 9].

In the present paper we report that RESV has a significant inhibitory effect on* C. trachomatis *propagation in McCoy cells.* C. trachomatis *is the most common sexually transmitted bacterial pathogen causing a wide range of urogenital diseases (urethritis, endocervicitis, salpingitis, endometritis, and inflammatory pelvic disease) as well as trachoma and pulmonary infections [10–12]. The search for new antichlamydial agents has become especially important due to the global increase in antibiotic-resistant chlamydial infections [10, 13].

## 2. Material and Methods

### 2.1. Reagents and Organisms

All reagents were from Sigma-Aldrich unless specified otherwise.* C. trachomatis *strain L2/Bu434 and* C. pneumoniae *strain Kajaani 6 were kindly provided by Dr. P. Saikku (University of Oulu, Finland).* C. muridarum *strain Nigg (ATCC VR-123). Resveratrol (RESV) was purchased from Kaden Biochemicals (Germany), dissolved in ethanol at a concentration of 50 mM and kept at −80°C as a stock solution.

### 2.2. Cell Culture

*C. trachomatis *and* C. muridarum *were propagated in McCoy mouse fibroblasts grown in DMEM with 10% HyClone FCS supplemented with 2 mM glutamine, 4.0 mg/ml gentamicin, and 5.0 mg/ml amphotericin B.

*C. pneumoniae *was propagated in HL cells (Washington Research Foundation, Seattle, USA).* C. trachomatis *was initially propagated in McCoy cells and purified by Renografin gradient centrifugation [14]. Elementary bodies were isolated and resuspended in SPG buffer. Titers were determined by infecting cell monolayers with 10-fold dilutions of thawed stock suspension. Subconfluent McCoy cell monolayers were infected with the stock suspension of* C. trachomatis *at a multiplicity rate of 1 : 1. Plates were centrifuged for 1 hour at 1500*g* to synchronize the infection. A wide range of RESV additions to the culture medium were performed at the “0” time point of the postinfection period.

Nucleic acid-based assays were performed 24 hours after* C*.* trachomatis *inoculation of the cultured cells. Infection rate in the McCoy cells and infective progeny formation were estimated 48 hours after pathogen introduction into the incubation medium.

All experiments were supplemented with negative (additions of RESV solvent) and positive controls (addition of 0.67 *μ*M azithromycin). This concentration of azithromycin was defined as minimum inhibitory concentration (MIC_AZ_) under culturing conditions used in our studies.

### 2.3. Determining RESV Toxicity in McCoy Cells

*In vitro *cytotoxicity was estimated by MTT [3-(4,5-dimethylthiazol-2-y1)2,5-diphenyltetrazolium bromide] test. Briefly, McCoy cells were plated in 96-well plates. After overnight attachment in RPMI with 10% FCS (37°C, 5% CO_2_) and medium replacement, addition of 10 *μ*l of RESV was performed (final concentration was 12.5–100 *μ*M). After 24 or 48 hours incubation, 20 *μ*l of MTT (5 mg/ml) was added to each well. The plates were incubated for an additional 4 hours, washed in PBS, and treated with 100 *μ*l of isopropanol. The optical density was measured at 540 nm with a reference wavelength of 630 nm using a Multiskan EX microplate photometer (Thermo Fisher Scientific, USA). RSV toxicity was also measured using the LIVE/DEAD® Viability/Cytotoxicity assay according to the manufacturer's instructions (Invitrogen, UK).

Moreover, a trypan blue exclusion test was performed routinely during all experiments. The acceptable level of cell viability was set at ≥90%.

### 2.4. Immunofluorescence Staining

Infected McCoy cells were grown for 48 hours on coverslips in 24-well plates. The cells were fixed with ice-cold methanol, permeabilized with 0.1% Triton X-100, and preblocked for 1 hour at 4°C with 1% bovine serum albumin in PBS. All monolayers were stained using FITC-conjugated monoclonal antibody against chlamydial lipopolysaccharide (NearMedic Plus, RF). Inclusion-containing cells were visualized using a Nikon Eclipse 50i microscope at ×1000 or lower magnification.

### 2.5. Assessment of Infective Progeny

Infective progeny accumulation was assessed in McCoy cell monolayers infected with* C. trachomatis *with or without additions of RESV. Infected cell monolayers were harvested 48 hours after bacterial inoculation and lysed by freezing/thawing. Serial dilutions of lysates were inoculated onto McCoy cells and plates were centrifuged for 1 hour at 1500*g*. The infected cells were visualized at 48 hours of the postinfection period using FITC-conjugated monoclonal antibody against chlamydial lipopolysaccharide (NearMedic Plus, RF).

#### 2.5.1. Modified Enzyme Immunoassay (ELISA)

A modified enzyme immunoassay was carried out using 96-well plates 48 hours after pathogen inoculation. In brief, the cells were fixed with ice-cold 72% ethanol, placed for 30 min at −20°C and treated with StabilZyme® HRP Conjugate Stabilizer (SurModics, USA). After washing and 1-hour incubation with monoclonal antibody against* C. trachomatis *MOMP (NearMedic Plus, RF), the cells were washed again with PBST and exposed to anti-mouse IgG conjugated with horseradish peroxidase. After additional PBS washing, the cells were incubated for 30 minutes with 0.3% 3,3,5,5-tetramethylbenzidine substrate and the reaction was stopped with H_2_SO_4_. The results were read in triplicate wells on a Multiskan EX at 450 nm.

### 2.6. Attachment and Internalization Assay

This assay was performed as described in our previous paper [15].

### 2.7. RNA Extraction and Reverse Transcription

RNA was isolated from* C. trachomatis-*infected McCoy cell monolayers grown on 6-well plates using TRIZol (Invitrogen) at the 24-hour time point of the postinfection period. Total mRNA was pretreated with DNase I (DNA-free™, Ambion) and quantified on a NanoDrop ND-100 spectrophotometer (Thermo Fisher Scientific, USA). 1 *μ*g of each RNA sample was converted into cDNA using random hexamer primers and a SuperScript III First-Strand Synthesis Kit (Invitrogen, Germany).

### 2.8. Quantitative Real-Time PCR

mRNA levels for three different developmental genes of* C. trachomatis *were analyzed in McCoy cells after treatment with RESV by quantitative RT-PCR using a CFX-96 thermocycler (Bio-Rad Laboratories, USA). RT-qPCR Taqman primers were designed using Primer 3 software and were validated by BLAST search as well as regression plot analysis using *C*_*p*_ values obtained with multiple dilutions of cDNA. Specificity of the designed primers and fluorescent probes was confirmed in different systems using cDNA from* C. trachomatis Bu-434*,* C. trachomatis UW-3/Cx, C. trachomatis UW- 31/Cx, *and* C. pneumoniae K-6*, as well as from uninfected McCoy cells and some other common bacterial pathogens.

The* C. trachomatis-*specific primers used were as follows:

- For* euo *gene: Pr-F 5′ TCCCCGACGCTCTCCTTTCA 3′, Pr_R 5′CTCGTCAGGCTATCTATGTTGCT 3′, Probe 5′- ROX- ATG GAC GCC ACT TGT CCC ACG GAA T- BHQ2-3′
- For* incA *gene: Pr*-*F 5′ CTACAGAAGAAATGCGCAAACTTT, Pr-R 5′ AATGATTGCTGGTTATGCGCTAAT, Probe 5′-FAM – CGGCGAACTTCTTCTGCTAATGGGGTT BH1- 3′
- For* omcB *gene: Pr-F 5′-TGTATCAGAAACTGGAACAGTCAATG 3′, Pr-F 5′TGAAAGCAGTATCAGCTGGAGATG 3′, Probe 5′-FAM CGGAAGAAAGAATCGCTTCCCCACG BH1- 3′

Primer sequences for eukaryotic beta-actin were used as published previously [15].

All primers were verified and used under thermal cycling conditions—95°C for 10 min and 50 cycles of 95°C for 15 seconds, 60°C for 1 min, and 72°C for 20 seconds. Serial dilutions of RNA, extracted from* C. trachomatis*-infected McCoy cells, were used as a standard for quantification of chlamydial gene expression. The mRNA expression levels were referenced to Ct values for chlamydial genes detected in infected McCoy cells grown at “0” concentration of RESV. This reference value was taken as 1.00. All mRNA measurements were done in triplicate.

All experiments were conducted at least three times. Statistical analysis was performed where possible using Student's *t*-test. The most representative sets of immunofluorescent images were selected and are shown above.

## 3. Results

### 3.1. Cytotoxicity Assessment

**Figure 1:**
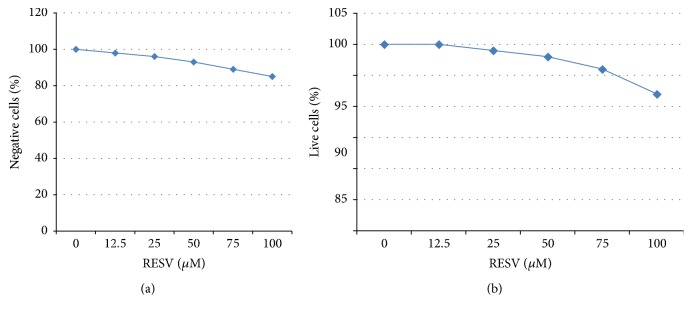
Assessment of cytotoxicity in McCoy cells incubated with increasing concentrations of resveratrol (RESV). McCoy cells were plated in 96-well plates. After overnight attachment and medium replacement, addition of 10 *µ*l of RESV was performed (final concentration was 12.5–100 *µ*M). After 24 hours incubation MTT test (a) and LIVE/DEAD Viability/Cytotoxicity assay (b) were performed as described in* Material and Methods*.

As can be seen from Figure 1, RESV showed no significant cytotoxicity in subconfluent McCoy cell monolayers when present in the incubation medium for 24 hours at a concentration of 12.5 *μ*M to 75 *μ*M (*P* > 0.05). However, the highest RESV concentration used (>100 *μ*M) produced a notable increase in the number of McCoy cells positive in MTT and Live/Dead assays. An acceptable level of cell viability (~90%) was also detected at later time points during McCoy cell incubation with 12.5–75 *μ*M RESV (up to 48 hours, results not shown) which allowed immunofluorescence studies to be carried out 48 hours after RESV additions. No significant effect of RESV on host cell proliferation was detected under the conditions used.

#### 3.1.1. Immunofluorescence and Enzyme Immunoassay Analysis

**Figure 2:**
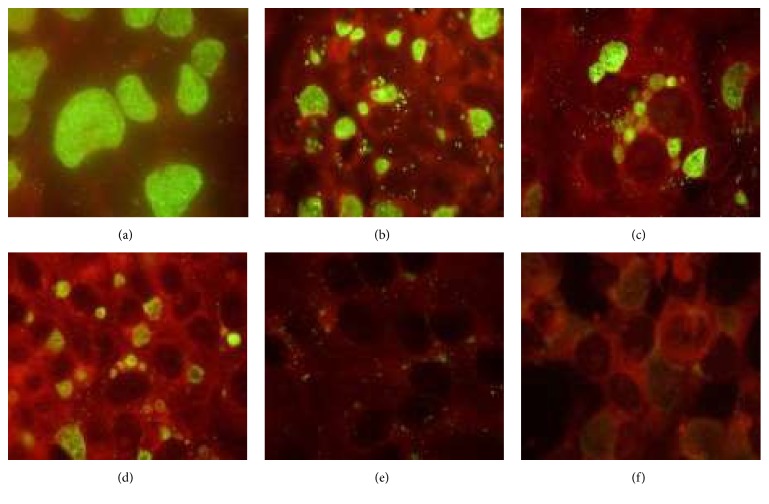
Resveratrol (RESV) inhibits propagation of* C. trachomatis* in McCoy cells. Immunofluorescence analysis (×1000). McCoy cells were infected with* C. trachomatis *serovar L-2 at MOI of 1. The final concentrations of RESV present in the culture medium ranged from 12,5 to 100 *µ*M, as indicated. Cultures were fixed after 48 hours incubation at 37°C in 5% CO_2_. Infected monolayers were stained using* C. trachomatis*-specific monoclonal antibodies as described in Material and Methods. (a) “0” *µ*M RSV, (b) 12.5 *µ*M, (c) 25 *µ*M, (d) 50 *µ*M, (e) 75 *µ*M, and (f) 100 *µ*M.

**Figure 3:**
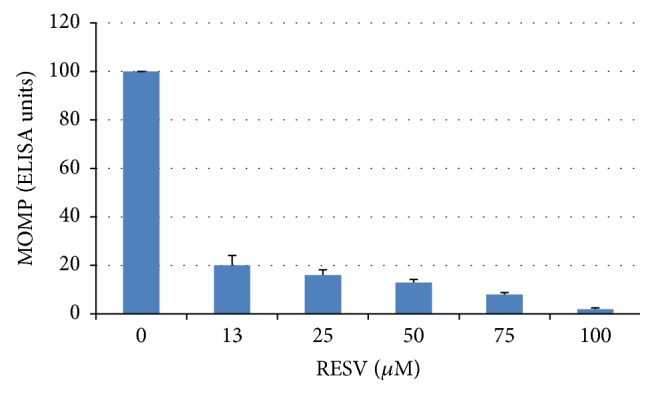
Relative amounts of MOMP in McCoy cells infected with* C. trachomatis *in the presence of resveratrol (RESV). McCoy cells were plated in 96-well plates, infected with* C. trachomatis *with simultaneous addition of RESV (0–100 *μ*M). At 48 hours after infection, infected monolayers were fixed, blocked, washed, and subjected to modified enzyme immunoassay (ELISA) with* C. trachomatis*-specific monoclonal antibodies against MOMP as described in* Material and Methods*.

As can be seen from Figure 2, simultaneous addition of RSV and the pathogen is accompanied by a dramatic inhibition of inclusion body formation in McCoy cells. Inclusion bodies in 48-hour McCoy cultures appeared to be large, polygonal, or round-shaped with homogeneous distribution of immunofluorescent signal. Even the lowest RESV concentration (12.5 *μ*M; Figure 2(b)) reduced the size of the inclusion bodies and their staining intensity. The inhibitory effect of RESV was even more apparent at a concentration 25 *μ*M when the number of inclusion bodies was reduced approximately twofold. There was an obvious “empty pocket” formation in the inclusion bodies at the 50–75 *μ*M RESV concentration range. Drastic reduction of inclusion body numbers was seen with 75 *μ*M RESV, while complete eradication of infection was achieved with 100 *μ*M RESV. No inclusion body formation has been seen at 100 *μ*M RESV. In order to quantify the RSV-induced changes in chlamydial growth in McCoy cells, immune detection of MOMP was performed. As shown in Figure 3, there was a stepwise decline in immune-detectable MOMP in McCoy monolayers infected with* C. trachomatis *in the presence of increasing concentrations of RESV. All experiments with RESV treatment were supplemented with a positive control (additions of 0.67 *μ*M azithromycin) which consistently showed a 100% reduction in the number of chlamydial inclusions without any significant toxicity for the McCoy cell monolayer in reference strain Bu434 of* C. trachomatis *under the conditions used in our cell culture experiments.

It has to be assumed that RESV arrests the infectious cycle at the stage of pathogen attachment/entry into the host cells. Hence, we decided to infect McCoy cells with RESV present in the medium (from 0 to 75 *μ*M) and document pathogen attachment to the cell membrane. According to our results, the presence of RESV in the medium had no noticeable impact on the attachment rate of EBs to the host cell membrane (data not shown).

#### 3.1.2. Infective Progeny Formation

**Figure 4:**
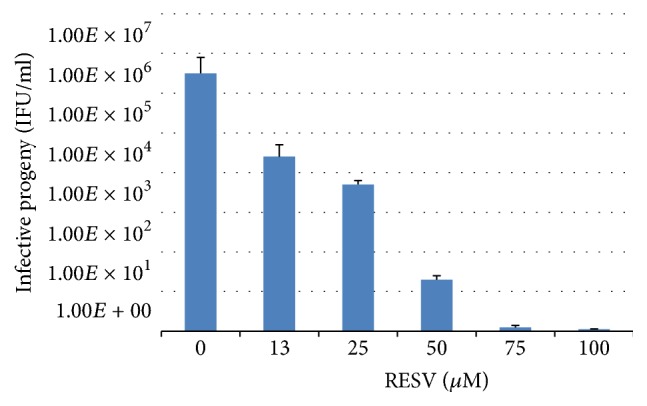
Infective progeny formation in McCoy cells infected with* C. trachomatis *in the presence of resveratrol (RESV). McCoy cells were plated, grown, and harvested at 48 hours after simultaneous addition of RESV (0–100 *μ*M) and* C. trachomatis. *Infective progeny formation was measured as described in* Material and Methods*.

Next, we decided to verify whether dose-dependent inhibition of chlamydial growth with RESV was accompanied by reduced formation of* C. trachomatis *infective progeny.  Figure 4 shows that there is a statistically significant and gradual decline in the infective progeny titer caused by additions of RESV in the concentration range of 12.5 *μ*M to 50 *μ*M. No infective progeny was detected at the highest RESV levels in the medium (75 *μ*M and 100 *μ*M).

Simultaneous addition of RESV and bacteria may arrest the infectious cycle by affecting the infectious properties of EBs. Therefore, in the following experiment we infected McCoy cells with bacterial particles pretreated with RESV and assessed* C. trachomatis *growth at 48 hpi using an IF protocol. The EBs of* C. trachomatis *were incubated for 1 hour at 37°C with increasing concentrations of RESV or vehicle alone, washed twice with SPG buffer, and inoculated into cultured cells. No statistically significant differences in percentage of infected McCoy cells were detected in these experiments (results not shown). Therefore, the RESV effect on* C. trachomatis *growth does not seem to be related to the direct effect of the compound on chlamydial progeny viability and infectivity.

#### 3.1.3. RNA Analysis

Finally, RNA analysis was employed to explore the inhibitory effect of RESV on* C. trachomatis *growth in McCoy cells. Biochemical and morphological abnormalities in the infected eukaryotic cells are secondary to transcriptional changes associated with the bacterial genome. Therefore, chlamydial mRNAs were measured at 24 hpi as opposed to the 48-hour time point used in our experiments for all other assays. As can be seen in Table 1, RESV treatment reduced significantly all mRNA values for genes representing the early, middle, and late phases of the chlamydial developmental cycle (e*uo*,* incA*, and* omcB*, resp.). Such a reduction was seen even with the lowest RESV concentration used (12.5 *μ*M) and reached a peak at 100 *μ*M. The only exception was* euo *mRNA. Its values showed a decline at 12.5 and 25 *μ*M RESV; however, higher RESV concentrations gave a less pronounced reduction in* euo *transcripts. Overall, the trend in chlamydial mRNA regulation in the presence of RESV was in good agreement with all of the other changes observed in* C. trachomatis*-infected McCoy cells treated with RESV.

#### 3.1.4. RESV Effect on* Chlamydiaceae*

The ability of RESV to inhibit other* Chlamydiaceae *was tested. McCoy cells were infected with* C. muridarum *and* C. pneumoniae *in the presence of RESV at concentrations from 12.5 to 100 *μ*М. Both of the* Chlamydiaceae *tested were completely inhibited by 75 *μ*М RESV treatment.

## 4. Discussion

*C. trachomatis *is an obligate intracellular epitheliotropic bacterium with a biphasic life cycle and is responsible for a variety of human diseases. In cultured cells and the human epithelium* C. trachomatis *can exist in two distinct morphological forms: the infectious elementary body (EB) and the noninfectious metabolically active reticulate body (RB). A typical chlamydial infection cycle begins with the entry of the EB into the host cell. Upon entry, the EBs differentiate into the RBs, which multiply and differentiate back to EB within the endosome (chlamydial inclusion). Newly formed EBs are usually released from the disrupted cells to initiate a new round of infection in the adjacent host cells [12, 16].

Despite the fact that chlamydial infection can be treated with antibiotics, growing antibiotic resistance and the frequent occurrence of persistent infections dictate the necessity of the search for new non-antibiotic inhibitors of chlamydial growth [17]. Various compounds with antichlamydial activity including Toll-like receptor agonists, metalloprotease inhibitors, and inhibitors of the bacterial type III secretion system have recently been identified [18–20]. These compounds exercise their antichlamydial activity either by direct effect on* C. trachomatis *viability and/or by modulating the host cell response. Host-directed therapeutic strategies become a new emerging reality in the treatment of chlamydial infections.

In the present paper, we report that resveratrol, an antifungal compound found in grapes and other plants, has a distinct ability to inhibit the* C. trachomatis *developmental cycle in McCoy cells, a classic cell line used for chlamydial research. This major conclusion is well documented by several lines of experimental evidence. First of all, there is a dose-dependent decline in the number of* C. trachomatis*-infected McCoy cells when treated with RESV, as revealed by IF staining with LPS-specific monoclonal antibodies. A similar tendency has been observed with another immunoassay utilizing MOMP-specific monoclonal antibody. Moreover, there was a significant stepwise reduction in the number of* C. trachomatis *infective progeny induced by increasing concentrations of RESV. Finally, the ability of RESV to arrest the infectious cycle of* C. trachomatis *in McCoy cells was confirmed by nucleic acid amplification protocol. Additionally, the inhibitory effect of RESV was reproduced for other chlamydial species including* C. pneumoniae *and* C. muridarum.*

Susceptibility assessment showed that the minimum inhibitory concentration (MIC) for RESV in* C. trachomatis*-infected McCoy monolayers is 75 *μ*M which is approximately 100 times higher than the corresponding value for azithromycin (0.67 *μ*M), an antibiotic widely used for treatment of chlamydial infections. However, the antichlamydial activity of RESV needs to be reassessed in further cell culture studies, animal experiments, and most importantly clinical studies. RESV is known to have low bioavailability rate in in vivo conditions owing to its high susceptibility to oxidation and the hydrophobicity of the molecule [21, 22]. Thus, it is very likely that “smart delivery” technologies (nanoparticles, nutraceutical formulations) may significantly enhance the antichlamydial effect of RESV.

Nevertheless, the newly discovered anti-infective properties of polyphenols, including RESV, mark an important development in modern pharmacotherapy of infectious diseases. Antiviral activity of RESV has recently been shown in Herpes simplex, varicella-zoster, and influenza viruses as well as human cytomegalovirus [23–25], whereas antimicrobial activity of RESV has only been reported for* Neisseria gonorrhoeae*,* Neisseria meningitidis, *and other bacterial pathogens [26].

The mechanisms behind the antiviral and antibacterial properties of RESV need to be thoroughly investigated. Although our results do not disclose the precise molecular mechanisms behind the antichlamydial activity of RESV, some conclusions can be drawn from our data. The antichlamydial activity of RESV is not related in our view to its potential toxicity to eukaryotic cells nor to RESV effect on host cell proliferation since it has been observed at a wide range of RESV concentrations proven to be nontoxic (12.5 *μ*M–75 *μ*M). Furthermore, according to our results, the inhibitory effect of RESV is not related to the potential effect of the compound on the attachment/entry of* C. trachomatis *to the host cells or to the direct effect of RESV on chlamydial particles. Although the exact mechanism of antichlamydial activity of RESV is yet to be determined, it is obvious that RESV affects an intracellular stage of the* C. trachomatis *infectious cycle. In our opinion, the results reported above may reveal the dependence of the chlamydial developmental cycle on the host cell sirtuin pathway, a family of NAD(+)-dependent deacetylases which are a primary target of RESV in eukaryotic cells. Therefore, therapeutic strategies targeting the sirtuin pathway of host cells exposed to chlamydial pathogens should be carefully evaluated in future studies. If our results obtained in cultured cells have some equivalence to* in vivo *systems, the addition of RESV to antibiotic regimens may hold some promise for treatment of chlamydiosis.

Moreover, there is an interesting pattern of chlamydial mRNA regulation in McCoy cells treated with RESV. An unequivocal and steep dose-dependent decline in mRNA values for* incA* and* omcB*, which are markers for the middle and late stages of the chlamydial developmental cycle, was accompanied by discordant changes in* euo *mRNA. The* euo *gene is a newly emerging informative marker of the early phase of the chlamydial developmental cycle which encodes a histone H1-specific protease [27]. Its mRNA values were significantly reduced at lower RESV concentrations (25 and 50 *μ*M). In contrast, higher concentrations of RESV (75 and 100 *μ*M) caused less significant euo mRNA reduction. If this pattern of euo mRNA changes is complemented by a relatively well preserved histone condensation rate, our results may suggest that the early phase of the chlamydial developmental cycle is less impacted by RESV treatment than the middle and late stages of* C. trachomatis *infection. Although such reasoning is highly speculative, further investigation focused on the middle and late stages of chlamydial infection may reveal important mechanisms behind the antichlamydial activity of RESV. Additional studies are required to verify whether RESV has some antichlamydial activity under “in vivo” conditions and whether our recent results have any relevance to clinical practice.

## Figures and Tables

**Table 1:** Folds and mRNA changes in McCoy cells infected with *C. trachomatis* in the presence of resveratrol (RESV).

mRNA		RESV concentrations (*μ*M)				
	0	12.5	25	50	75	100
*euo*	1	0.2 ± .0.05^*∗*^	0.05 ± 0.075^*∗*^	0.033 ± 0.013^*∗*^	0.35 ± 0.03^*∗*^	0.315 ± 0.065^*∗*^
*incA*	1	0.175 ± 0.075^*∗*^	0.09 ± 0.01^*∗*^	0.055 ± 0.015^*∗*^	0.07 ± 0.01^*∗*^	0.064 ± 0.005^*∗*^
*omcB*	1	0.059 ± 0.001^*∗*^	0.04 ± 0.01^*∗*^	0.025 ± 0.005^*∗*^	0.017 ± 0.002^*∗*^	0.015 ± 0.005^*∗*^

McCoy cells were set up, grown, and infected with *C. trachomatis *in the presence or absence of increasing concentrations of RESV (0–100 *μ*M) as described in Material and Methods. Total RNA was extracted 24 hours later. RNA levels for the genes of interest were normalized to eukaryotic *β*-actin expression levels. All mRNA values were referenced to expression levels at “0” RESV concentration (1.00); *∗* indicates statistically significant values as compared to “0” RESV concentration.
